# Estimation of Forces and Powers in Ergometer and Scull Rowing Based on Long Short-Term Memory Neural Networks

**DOI:** 10.3390/s25010279

**Published:** 2025-01-06

**Authors:** Lorenzo Pitto, Frédéric R. Simon, Geoffrey N. Ertel, Gérome C. Gauchard, Guillaume Mornieux

**Affiliations:** 1 Development Adaptation Handicap (DevAH) Research Unit, Université de Lorraine, 54000 Nancy, France; lorenzo.pitto@univ-lorraine.fr (L.P.); frederic.simon@univ-lorraine.fr (F.R.S.); geoffrey.ertel@univ-lorraine.fr (G.N.E.); gerome.gauchard@univ-lorraine.fr (G.C.G.); 2 CARE Grand Est, Université de Lorraine, 54000 Nancy, France; 3 Faculty of Sport Sciences, Université de Lorraine, 54000 Nancy, France

**Keywords:** machine learning, rowing, IMU, kinetics, LSTM

## Abstract

Analyzing performance in rowing, e.g., analyzing force and power output profiles produced either on ergometer or on boat, is a priority for trainers and athletes. The current state-of-the-art methodologies for rowing performance analysis involve the installation of dedicated instrumented equipment, with the most commonly employed systems being PowerLine and BioRow. This procedure can be both expensive and time-consuming, thus limiting trainers’ ability to monitor athletes. In this study, we developed an easier-to-install and cheaper method for estimating rowers’ forces and powers based only on cable position sensors for ergometer rowing and inertial measurement units (IMUs) and GPS for scull rowing. We used data from 12 and 11 rowers on ergometer and on boat, respectively, to train a long short-term memory (LSTM) network. The LSTM was able to reconstruct the forces and power at the gate with an overall mean absolute error of less than 5%. The reconstructed forces and power were able to reveal inter-subject differences in technique, with an accuracy of 93%. Performing leave-one-out validation showed a significant increase in error, confirming that more subjects are needed in order to develop a tool that could be generalizable to external athletes.

## 1. Introduction

Rowing is a sport that requires the precise coordination of all the body segments, making technique a determinant factor for performance, with the result of a race possibly being decided by differences as small as a fraction of a second [1,2]. Rowing involves cyclic movements, and each cycle is usually decomposed into two phases, namely, the drive and recovery phases. The ‘drive’ phase begins when the oars are inserted into the water; this event is called the ‘catch.’ During the drive, the oars remain immersed in the water and propel the boat forward [1]. During this phase, the sequential movement of the legs, trunk, and arms generates power that is ultimately transferred to the water through the oars [1]. At the end of this movement, the oars are extracted from the water, marking the event called ‘finish’ and the beginning of the ‘recovery’ phase. During this phase, the oars remain out of the water, and the rowers pull the boats with their feet in order to prepare for the following cycle. When weather conditions are unsuitable for rowing on water, rowers often train indoors using rowing ergometers [3]. Some ergometers have been designed to reproduce as closely as possible the forces and movements of boat rowing by including a free-floating mechanism that allows the frame of the ergometer to slide in the horizontal direction [3].

In recent years, significant efforts have been made to precisely measure and quantify the rowing technique (commonly referred to as the “athlete signature” [4]) and performance through the integration of motion and force sensors on boats and ergometers [4,5,6,7,8,9,10,11,12]. Force and power profiles are of particular interest because they correlate with performance [1,13] and the spinal loads associated with low back pain, which has a high prevalence among rowers [14,15]. The analysis of force–time profiles is also used as a criterion for selecting crew members in double and four boats [1,16]. Additionally, the decomposition of the total power generated by the rowers into the contributions of the different body segments (i.e., arms, trunk, and legs) is a powerful tool that can be used to characterize a rower’s technique and, thus, identify and correct technical errors [1,17,18]. To quantitatively describe these force and power profiles, technical determinants, such as the time to peak (T2P), mean to peak ratio (M2P), and work ratio (WR), are typically used. More uniform force profiles, characterized by a higher M2P [12,13], as well as an earlier peak in the force transferred to the oars (i.e., a lower T2P) [9,12], have been associated with higher performance. Furthermore, an earlier peak of maximum trunk power has been associated with higher performance [17], a factor that can be directly targeted by using real-time biofeedback to improve a rower’s technique [18].

**Figure 1:**
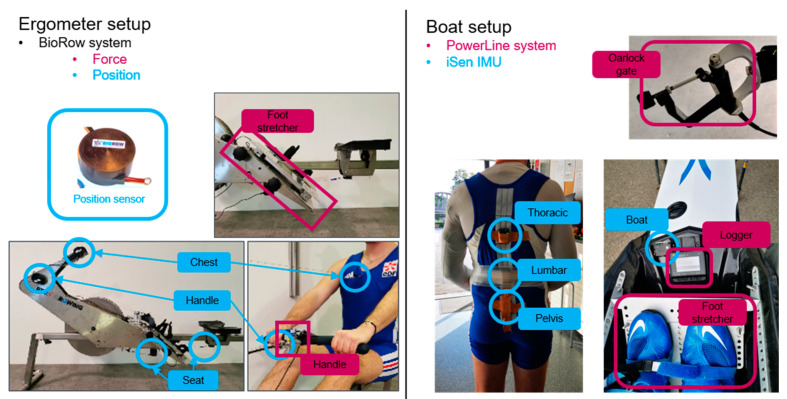
Sensor setup for ergometer and boat rowing.

While measuring the forces generated by rowers provides important information to trainers, it requires the replacement of certain components of a boat or ergometer with specialized, instrumented versions. Two of the most commonly used systems for analyzing rowing performance are BioRow (BioRow Tech, London, UK) and PowerLine (Peach Innovation, Cambridge, UK). The proposed solutions (Figure 1) involve replacing the foot stretcher (the component that connects to the rowers’ feet, allowing the transfer of pushing and pulling forces) in both boat and ergometer rowing, replacing the oarlocks at the boat gates (components of the boat that hold the oars in place, allowing them to pivot during the rowing stroke), and adding a strain gauge at the handle in ergometer rowing (on an ergometer, the oars are replaced by a single rigid handle attached to a chain) [4,5,8,12,19]. This type of equipment can be expensive, and significant effort is required to keep the same rigging when parts are replaced, especially in scull rowing, where boats are finely adjusted according to the anthropometry and preferences of each rower [1]. These factors inevitably limit the number of boats or ergometers that can be equipped simultaneously and the frequency at which the instrumented parts can be installed on different boats, which, in turn, limits the amount of attention paid by trainers to each rower. A method allowing the estimation of the forces and powers generated by rowers without necessitating the replacement of boat or ergometer parts would be extremely helpful, allowing multiple rowers within a club to be quickly equipped and monitored.

Machine learning methods have been widely applied to the detection of human behavior, particularly within the field of biomechanics. These applications range from the classification and detection of specific features of motion, like gait abnormalities [20,21] and swimming styles [22], to the estimation of joints [23], bones [24], and ground reaction forces [25]. Recently, companies have started developing machine learning methods based on physics-informed biomechanical simulations for estimating body internal and contact forces from videos [26,27]. When applied to rowing practice, machine learning methods have been used to cluster rowers based on kinematics [28], separate novice from expert rowers based on posture measured with inertial measurement units (IMUs) [29], and assign rowers to specific events based on kinematics and demographics [30].

An alternative to machine learning is the use of traditional biomechanical simulations. In particular, predictive and tracking simulations have been successfully employed to study human motion [31,32,33] and are well suited for estimating forces and power output in rowing applications. Tracking simulations execute forward dynamics while forcing the motion to closely match the recorded kinematics, enabling the generation of dynamically consistent kinematics and kinetics even in the absence of measured forces [31]. However, this approach suffers from the drawback of being more computationally demanding and requiring expert users to handle the different aspects of the simulation, such as defining models that describe muscle architecture and force generation, as well as interactions with the environment.

To the best of our knowledge, there are currently no machine learning methods for estimating the force and power generated by rowers, and the gold standard for rowing performance analysis remains the post-session analysis of PowerLine or BioRow data. This paper contributes to the state of the art by providing a tool for estimating rowing forces and powers based on measured kinematics.

Our main goal was to estimate the time profiles of force and power throughout the rowing cycle rather than clustering rowers based on technique [28] or using classifiers to define their performance [29]. Given the sequential nature of the rowing movement, characterized by time-dependent variations in forces and a sequential involvement of the body segments, we chose to base this work on a recurrent neural network. Long short-term memory (LSTM) networks are particularly well suited for processing time series information and have been extensively employed for sequence-to-sequence tasks in various applications for several years [34], including in biomechanics tasks, to recognize motor activities [34], predict falls [35], gait kinematics [36,37], and gait phases [36]. The functioning of LSTM networks has been described exhaustively in many publications [37,38,39,40] and is beyond the scope of this paper. In short, the architecture of LSTM networks allows them to capture long-term relationships within sequences, with the ability to selectively retain or forget information [40].

The input data for the proposed model should come from sensors that are cost-effective and easy to install. When rowing on an ergometer, cable-based sensors can be used to measure the displacement of different body parts [17,18]. These sensors consist of a spring-loaded cable that can be easily attached and detached to a rower’s clothes or ergometer seat. On a boat, the most versatile method for measuring rowers’ movements involves the use of IMUs. IMUs are compact and lightweight sensors that can be comfortably worn by rowers without restricting their movement and installed on the boat without any alteration of the rigging. Thanks to their versatility, IMUs have been extensively used to measure human motion [32,41,42,43].

The purpose of this study was to train an LSTM network to determine the forces and powers generated by rowers during boat and ergometer rowing without the use of force sensors. In particular, we aimed at computing the forces at the feet and gates or handle, the power transferred to the gates or handle, and the powers generated by the arms, trunk, and legs. We hypothesized that the accuracy of force and power estimation would be enough to describe the rowers’ techniques, characterized by features such as peak timing and the development ratio.

## 2. Materials and Methods

### 2.1. Participants

Data for this study were collected as part of previously published studies [12,18]. This included data from 12 male rowers on a scull boat (20.0 ± 2 years old, 185 ± 5 cm, 79.0 ± 6.7 kg) and from 11 male rowers on the ergometer (22.3 ± 2.2 years, 185.5 ± 7.1 cm, 77.4 ± 7.0 kg). Participants did not suffer any recent injury.

### 2.2. Test Procedure

Concerning the dataset collected from rowing on water, rowers were asked to row at their personal competition rate, which was, on average, 32.5 strokes per min (spm), and used their personal scull boat. The dataset on the ergometer included rowing sessions with two different stroke rates, i.e., 20 spm and their personal competition rate, which was, on average, 35.8 spm.

### 2.3. Setup and Data Acquisition

The ergometer (RP3^®^, Care RowPerfect BV, Hardenberg, The Netherlands) was equipped with BioRow sensors. These included force sensors at the feet and handle and displacement sensors at the handle, seat, and chest (Figure 1). The position sensors consisted of spring-loaded cables. These sensors were connected to an acquisition card (National Instruments, “NI USB-6000 Multifunction I/O Device,” National Instruments, Austin, TX, USA) connected to Matlab (MATLAB, version R2022b, The MathWorks, Inc., Natick, MA, USA) and sampled at 150 Hz. The ergometer was utilized in its dynamic configuration, and all measurements were performed at the same wheel resistance (i.e., 5 on the RP3 wheel).

The personal scull boats of the rowers were equipped with a PowerLine system (version 4.12.0.0). This system included force sensors at the foot stretcher and oarlock gates and oar angle sensors at the oarlock gates (Figure 1). The sensors were connected to a logger unit positioned in front of the rowers, which also collected data from an integrated GPS and IMU and provided real-time feedback, such as stroke rate and power output. PowerLine data were collected at 50 Hz on the logger and downloaded after the rowers finished their training session. Additionally, four IMUs (version 2022.0, iSen, STT Systems, San Sebastian, Spain), collecting data at 100 Hz, were included: three on the back of the rower (pelvis, lumbar, and thoracic spine) [42] and one on the boat to facilitate synchronization with the PowerLine data. These IMUs are compact and lightweight (46 g, 56 mm × 38 mm × 18 mm), minimizing interference with the rowing motion. They incorporate built-in sensor fusion algorithms that enable direct export of the unit 3D pose and free acceleration data. Data from IMUs were wirelessly transmitted to a receiver placed on a boat closely following the athletes. Given this limitation, iSen data sequences were limited to a series of around 15 consecutive strokes and were, therefore, much shorter than the ones stored on the PowerLine logger.

### 2.4. Data Analysis

Data from the iSen and PowerLine systems were synchronized based on the boat velocity measured from the PowerLine GPS and the boat velocity computed from the acceleration of the iSen IMU installed on the boat. Specifically, the IMU acceleration measured along the direction of movement was time-integrated and high-pass filtered (0.1 Hz) to remove drift; the velocity measured by the GPS was similarly filtered. The points of minimum velocity were automatically identified and used to determine rowing cycles and the instantaneous stroke rate. This allowed the identification of the cycles recorded during the short iSen sequences within the much longer PowerLine sequences. After synchronization, all data from the boat rowing sessions were interpolated at 100 Hz.

Rowing cycles were segmented based on the starboard position of the oar for scull rowing and on the handle position for ergometer rowing. In both cases, the lowest value defined the catch event, and the highest value the finish event. After discarding the cycles with missing data and artifacts, the datasets consisted of 396 cycles for boat rowing and 2005 cycles for ergometer rowing.

In both rowing conditions, we considered only the force along the axis of motion (defined by the boat axis and the ergometer rail). This translates to the horizontal component of the foot stretcher force. Similarly, for the force measured at the oarlock gates, only the propulsive component, along the direction of motion, was considered. For the force at the ergometer handle, the installed strain gauge only measured a unidirectional force, specifically the tension of the chain.

The cable sensors installed on the ergometer measured the position of the handle, chest, and seat with respect to the ergometer ($X_{ergo}^{h}$, $X_{ergo}^{c}$, and $X_{ergo}^{s}$, respectively). The length of the arms ($X_{ergo}^{a}$) and the trunk opening ($X_{ergo}^{t}$) were computed as:

(1) $$X_{ergo}^{a}=X_{ergo}^{h}-X_{ergo}^{c},$$

(2) $$X_{ergo}^{t}=X_{ergo}^{c}-X_{ergo}^{s},$$

**Figure 2:**
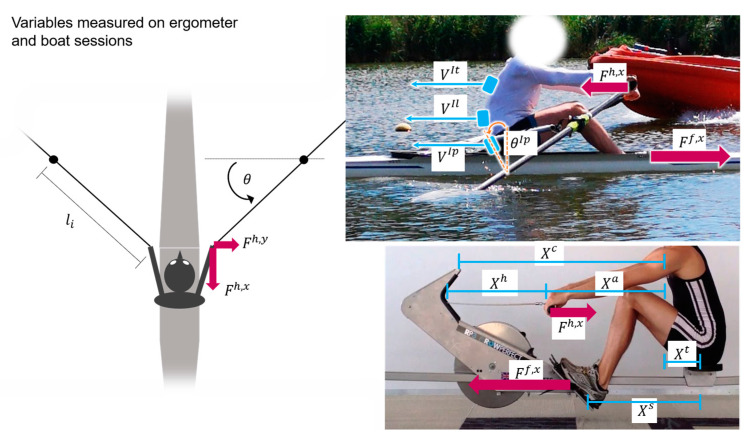
Graphical representation of the base variables included in the study.

The leg extension was assumed to be the same as $X_{ergo}^{s}$. The velocities of the handle and the three body segments $V_{ergo}^{h}$, $V_{ergo}^{a}$, $V_{ergo}^{t}$, and $V_{ergo}^{l}$ were obtained by taking the time derivatives of $X_{ergo}^{h}$, $X_{ergo}^{a}$, $X_{ergo}^{t}$, and $X_{ergo}^{s}$, respectively (Figure 2).

For the ergometer, power at the handle and segments was computed following Kleshnev [44]. Specifically:

(3) $$P_{ergo}^{h,x}=V_{ergo}^{h}F_{ergo}^{h,x},$$

(4) $$P_{ergo}^{a}=V_{ergo}^{a}F_{ergo}^{h,x},$$

(5) $$P_{ergo}^{t}=V_{ergo}^{t}F_{ergo}^{h,x},$$

(6) $$P_{ergo}^{l}=V_{ergo}^{l}F_{ergo}^{f,x},$$

where $P_{ergo}^{h,x}$, $P_{ergo}^{a}$, $P_{ergo}^{t}$, and $P_{ergo}^{l}$ are the powers for the handle, arms, trunk, and legs, respectively; $F_{ergo}^{h,x}$ is the force measured at the handle, and $F_{ergo}^{f,x}$ is the force measured at the feet along the horizontal direction.

For the boat, we computed the corresponding powers. However, given the differences in the systems and sensors, we used a different approach. In particular, the total power transferred to the oars ($P_{boat}^{h}$) was computed as:

(7) $$P_{boat}^{h}={(F}_{boat}^{h,x}l_{i}cos\left(\theta \right)+F_{boat}^{h,y}l_{i}sin\left(\theta \right))\dot{\theta }=P_{boat}^{h,x}+P_{boat}^{h,y},$$

where $l_{i}$ is the internal lever arm of the oar; $F_{boat}^{h,x}$ and $F_{boat}^{h,y}$ are the x and y components of the force at the handle; θ and $\dot{\theta }$ are the oar angle and angular velocity, respectively. Forces and powers at the handle are the sum of the board and starboard components.

The powers of the trunk and leg segments were computed as:

(8) $$P_{boat}^{l}=F_{boat}^{f,x}V_{boat}^{l},$$

(9) $$P_{boat}^{t}=F_{boat}^{h,x}V_{boat}^{t},$$

where $V_{boat}^{t}$ and $V_{boat}^{l}$ are the velocities of the trunk and leg segments:

(10) $$V_{boat}^{t}=V_{boat}^{It}V_{boat}^{Ip},$$

(11) $$V_{boat}^{l}=V_{boat}^{Ip}-l^{Ip}{\dot{\theta }}^{Ip}cos\left(\theta^{Ip}\right),$$

where $V_{boat}^{It}$ and $V_{boat}^{Ip}$ are the velocities of the IMUs placed on the thoracic spine and pelvis, obtained by time-integration of the IMUs acceleration; $l^{Ip}$ is the distance from the pelvis IMU to the seat (we used a value of 15 cm for all subjects); $\theta^{Ip}$ and ${\dot{\theta }}^{Ip}$ are the angle of the pelvis IMU from the vertical axis and its time-derivative.

The calculation of the power arms was based on Hofmijster et al. [45], proposing that the total power generated by the rower should be determined by the power transferred to the oar plus the power used to accelerate the center of mass. Modeling the rower’s body with only three segments capable of generating power, we obtain the power generated by the arms as:

(12) $$P_{boat}^{a}=P_{boat}^{h}+(F_{boat}^{f,x}-F_{boat}^{h,x})V_{boat}-P_{boat}^{t}-P_{boat}^{l},$$

where $V_{boat}$ is the boat velocity obtained from the GPS embedded in the PowerLine box.

In summary, the sequences used as targets for the LSTM network, hereafter referred to as ‘measured sequences,’ were: $F^{f,x}$, $F^{h,x}$, $P^{h,x}$, $P^{a}$, $P^{t}$, $P^{l}$, for both ergometer and boat rowing.

### 2.5. LSTM Architecture

For this study, a vanilla LSTM [40] was chosen and implemented using the built-in functions of the Deep Learning Toolbox in MATLAB. Preliminary analyses indicated that adding extra LSTM layers did not significantly improve performance while increasing training time. Consequently, the random search was conducted using a single LSTM layer. The base architecture was defined as:

- Sequence input layer (nI)
- Fully connected layer (nI)
- LSTM layer
- Dropout layer (DOp)
- Fully connected layer (nO)
- Regression layer

Where nI is the number of input features (18 for the boat and 16 for the ergometer, Table 1); DOp is the dropout percentage; and nO is the number of output sequences (six for both ergometer and boat sessions). We performed a random search to find the best values of the following hyperparameters: number of units within the LSTM layers (log-uniform sampling between 10 and 500) and dropout percentage (0%, 5%, and 10%). To select the best architecture after the random search, we based the decision on the mean absolute error (*MAE*) computed over the effective drive phase (refer to the session LSTM performance evaluation).

### 2.6. LSTM Training

We performed the first training during the random search and then used the so-defined architecture for a leave-one-out validation (similar to the method used by Su et al. [36]). For the random search training, we split the data into 80% training and 20% validation. For the leave-one-out validation, we used N-1 subjects as training and the remaining subject as validation, repeating the process N times. We set a stopping criterion to the training if 20 epochs [46] elapsed without improvement in the validation data reconstruction, measured as the root mean squared error (RMSE) over the rowing cycle [36]. The parameters used for the training included the following: max epochs: 10,000; learning rate: 0.001; minibatch size: 32; Adam optimization algorithm. Boat and ergometer sessions LSTM were trained separately.

### 2.7. LSTM Data

For both the ergometer and boat, the target series were six sequences describing: $F^{f,x}$, $F^{h,x}$, $P^{h,x}$, $P^{a}$, $P^{t}$, $P^{l}$. For both the ergometer and boat, the sequences defined were segmented into rowing cycles starting at the finish event and ending at the following finish event. Rowing cycles were considered in absolute time rather than being time-normalized. Before training, data were normalized to the mean and standard deviation. The selection of input features was different for the ergometer and boat, as data were obtained with different kinds of sensors and described two systems that differ in terms of body movement and interactions with the environment. Specifically, for the ergometer sessions LSTM, we included 16 features: position of handle, chest, and seat ($X^{h}$, $X^{c}$, and $X^{s}$); velocities of the handle, arms, trunk opening, and leg extension ($V^{h}$, $V^{a}$, $V^{t}$, and $V^{l}$); acceleration of the trunk and legs, computed by the time derivative of the respective velocities. Additional features were added to include information about the subjects and the rowing session: height and weight of the rower, duration of previous and current drive phases, maximum handle speed of the previous stroke, and time elapsed between the maximum speed and the catch event of the current stroke. The reason behind the last two is that the resistance felt at the handle depends on the spinning velocity of the wheel, which decreases during the recovery phase when no force is applied to it. Thus, the time elapsed from the maximum should provide information about the wheel spinning velocity at the start of the drive phase. For the boat sessions LSTM, we included 18 features: gate angular velocity ($\dot{\theta }$); boat horizontal velocity (from the GPS integrated into the PowerLine box) and acceleration (from the accelerometer integrated into the PowerLine box); horizontal velocity with respect to the boat reference system of IMU thoracic, lumbar, and pelvis ($V_{boat}^{It}$, $V_{boat}^{Il}$, and $V_{boat}^{Ip}$); first principal component of acceleration and angular velocity of the three IMUs. Similarly to the ergometer, additional features were added to include information about the subjects and the rowing session: height and weight of the rower; average and maximum boat velocity in the previous cycle (providing information about the change in speed of the boat and thus to the power transferred to the oars); duration of the previous and current drive phases (providing information about the stroke rate, which is known to correlate with power output).

### 2.8. LSTM Performance Evaluation

To compare the performances of the networks, we used metrics computed over the effective drive phase duration, as there are little to no forces and powers during the recovery phase, making it of little value for performance evaluation. The effective drive phase, for both ergometer and boat rowing, was automatically detected within each cycle based on the handle force, as it is usually computed with the Peach software. Specifically, the phase began when the gait force exceeded 20 kg and ended when the force decreased below 10 kg. Although sessions at different stroke rates on the ergometer were included in the LSTM training to improve the generalizability of the reconstruction, only the sessions at 20 spm were considered for the subsequent performance analysis, as this condition included the highest numbers of consistent cycles. As a measure of performance, we considered the mean absolute error (*MAE*) between the target and reconstructed sequences (i.e., $F^{f,x}$, $F^{h,x}$, $P^{h,x}$, $P^{a}$, $P^{t}$, $P^{l}$) for all cycles and the *MAE* over the average curve over multiple cycles (*cMAE*). As each cycle input sequence was of a different duration, we interpolated the reconstructed sequences over 50 timeframes describing the effective drive phase.

(13) $$MAE=\frac{1}{CT}\sum_{c=1}^{C}\sum_{t=1}^{T}\left|y_{t}^{c}-{\hat{y}}_{t}^{c}\right|,$$

(14) $$cMAE=\frac{1}{CT}\sum_{t=1}^{T}\left|\sum_{c=1}^{C}\left(y_{t}^{c}-{\hat{y}}_{t}^{c}\right)\right|,$$

where *T* = 50 timeframes; *C* is the number of cycles included in the validation set. During leave-one-out validation, *C* refers to all of the cycles associated with a single subject. *MAE* and *cMAE* were also expressed as a percentage of the associated curve:

(15) $${(c)MAE}_{norm}=\frac{(c)MAE}{mean(\left|V\right|)},$$

where V is any of the time-normalized target variables.

We evaluated how technical determinants used to describe and evaluate a rower’s technique were affected by the process of estimation. Specifically, we calculated time to peak (T2P), defined as the instant at which the curves reach the peak, expressed in percent of the entire drive phase (i.e., catch to finish); mean to peak (M2P), defined as the ratio between the mean and maximum value of a curve times 100, over the entire drive phase; and work ratio (WR), defined as the ratio of the area under the curve before the peak to the total area under the curve times 100, also over the entire drive phase [17]. To determine whether the rowers’ technique could be accurately described from the estimated sequences, we performed two-sample *t*-tests (ttest2 function in MATLAB, with significance level 0.05) comparing the means of the technical determinants calculated from the measured sequences with those derived from the estimated sequences. We also assessed whether the technical determinants derived from the estimated sequences could distinguish between the techniques of different subjects, specifically in cases where the technical determinants derived from the measured sequences of two subjects showed significant differences. This was conducted by performing two-sample *t*-tests to compare the mean values of the technical determinants of each pair of rowers. Inter- and intrasubject *t*-tests were performed only on data from ergometer sessions since the number of cycles for each subject in the boat sessions was too small in some cases.

For the intrasubject differences, we computed the accuracy:

(16) $$As=\frac{HA}{HA+HR}*100,$$

where *HA* denotes the subjects for which the null hypothesis of equal mean could not be rejected (*p*-value > 0.05) and *HR* the subjects where the null hypothesis was rejected.

For the inter-subject differences, we computed the accuracy:

(17) $$Ap=\frac{CH}{CH+DH}*100,$$

where *CH* denotes subject pairs where the null hypothesis of the two subjects having equal mean for the analyzed technical determinant was similarly rejected or accepted when conducting the test on the measured and LSTM curves; *DH* denotes the subject pairs where there was no agreement on the null hypothesis rejection.

## 3. Results

**Figure 3:**
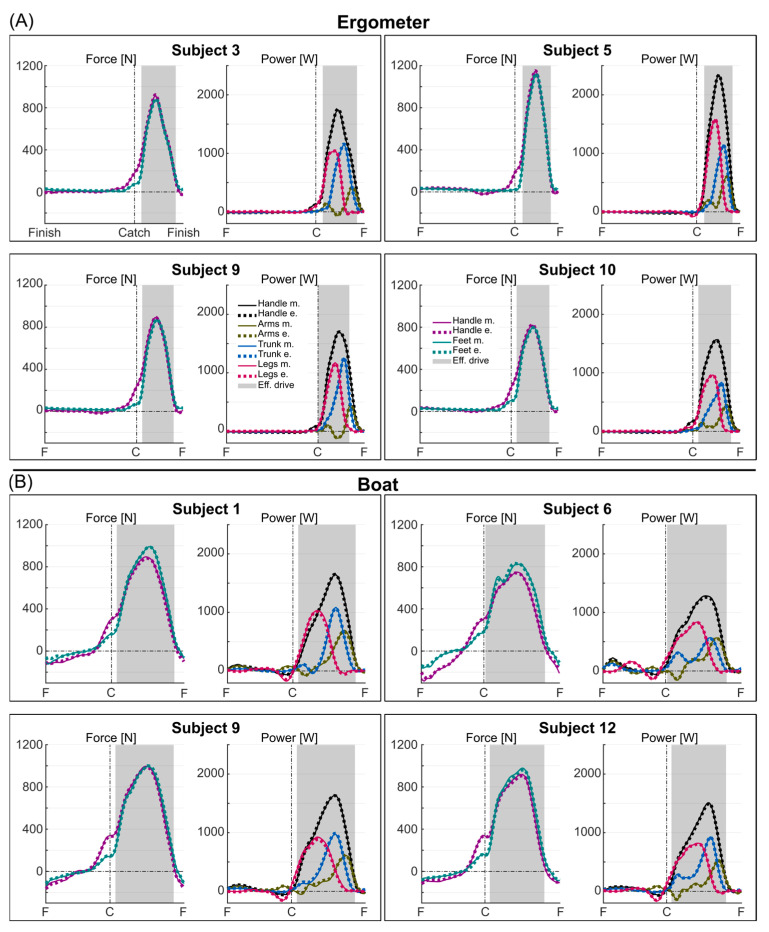
Measured (continuous lines) and estimated (dashed lines) force and power profiles for four representative subjects during ergometer (**A**) and boat (**B**) rowing. The curves represent the average over the cycles used for validation, time normalized over the finish-to-finish period. The vertical line defines the catch event; the shaded area defines the effective drive phase.

**Figure 4:**
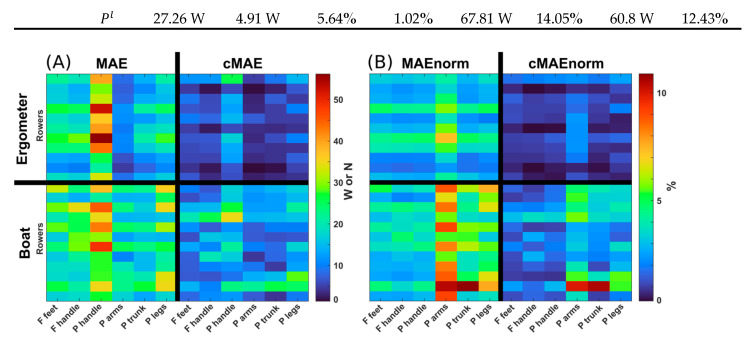
*MAE* and *cMAE* are reported in absolute values (**A**) and normalized to the average value of the target curve (**B**). Units are N for the forces (F) and W for the powers (P). Rows correspond to individual rowers.

The optimized architectures were found to be 1 layer, 500 neurons, and 0% dropout for the ergometer condition and 1 layer, 53 neurons, and 10% dropout for the boat condition. The average measured and estimated curves for the forces and powers of four representative subjects for the ergometer and boat sessions are depicted in Figure 3. The performance for all subjects combined, expressed in terms of absolute and normalized *MAE* and *cMAE* for all force and power variables, is presented in Table 2. The average values of *MAE_norm_* were 3.20% and 4.90% for ergometer and boat sessions, respectively; the average values of *cMAE_norm_* were 0.53% and 0.90% for ergometer and boat sessions, respectively. The *MAE_norm_* observed in the leave-one-out validation was on average 19.94% and 14.02% for the sessions on ergometer and on boat, respectively (Table 2); the *cMAE_norm_* was on average 7.95% and 12.38% for the ergometer and boat sessions, respectively. Figure 4 shows the performance metrics detailed subject by subject. The variable with the highest *MAE* and *cMAE* was $P^{h,x}$, and the variable with the highest *MAE_norm_* and *cMAE_norm_* was instead $P^{a}$.

**Figure 5:**
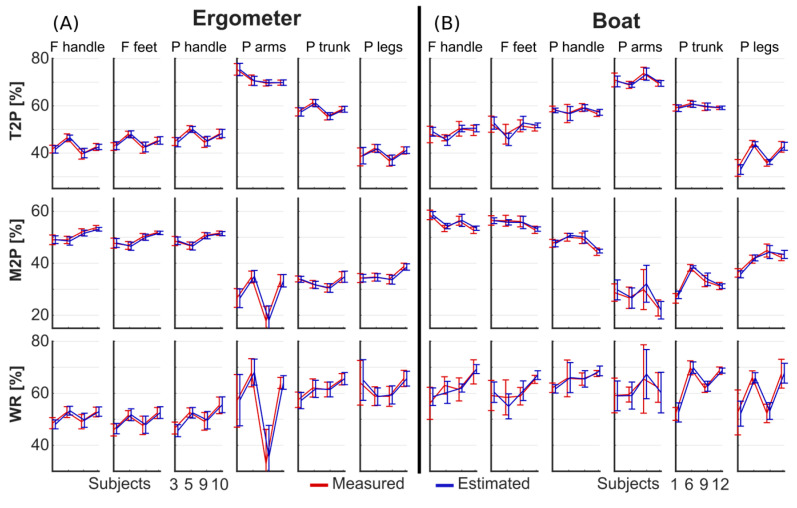
Technical determinants T2P, M2P, and WR were computed on the measured (red) and reconstructed (blue) forces and power sequences for four representative subjects in ergometer (**A**) and boat (**B**) rowing sessions. Mean and standard deviation were calculated across all cycles in the validation dataset.

**Figure 6:**
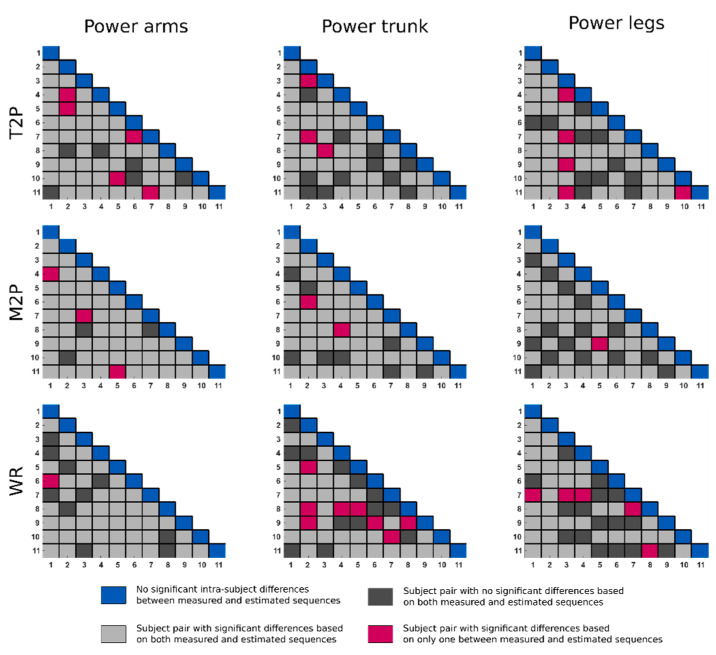
Two-sample *t*-test results between the measured and LSTM-estimated sequences for the T2P, M2P, and WR derived from the power arms, trunk, and legs. Values along the diagonal represent the intra-subject differences between measured and reconstructed sequences. Values outside the diagonal are comparisons between pairs of subjects performed on both the measured and estimated sequences. The colors indicate whether the inter-subject differences detected from the estimated sequences are consistent with those detected from the measured sequences.

The technical determinants describing the rowers’ technique (i.e., T2P, M2P, and WR) are shown in Figure 5 for four representative subjects. In both boat and ergometer rowing, a clear sequencing of legs, trunk, and arms was observed, with T2P occurring at approximately 40%, 60%, and 70% of the cycle, respectively. Subjects rowing on ergometers showed a variety of strategies regarding the involvement of the arms, as seen by the inter-subject variations in M2P and WR. Subjects on the boat showed more inter-subject variability in the involvement of the trunk and legs. The overall *MAE* of the T2P was 0.78% and 1.55%; the overall *MAE* for M2P was 0.66% and 0.96%; the overall *MAE* for WR was 2.05% and 3.32% for the boat and ergometer sessions, respectively (Table 3). When focusing on the ergometer sessions, no significant differences between T2P, M2P, and WR, based on the LSTM curves and on the measured curves, were found (Figure 6). When comparing pairs of subjects, technique differentiation for T2P for the power of the arms, trunk, and legs showed an accuracy of 90.9%, 94.5%, and 90.9%, respectively. These accuracies were 94.5%, 96.4%, and 98.2%, respectively, for the M2P; 98.2%, 85.4%, and 90.9% when based on the WR of the different segments.

## 4. Discussion

In this article, we propose a method for estimating the forces and powers generated during rowing with minimal setup and without requiring the installation of force sensors. The goal was to create a simple method that could be used by trainers during everyday practice and possibly serve as the basis for real-time feedback for rowers. In contrast, the use of traditional biomechanical approaches [31,33] would require more complex simulations, requiring expert users and more time-consuming analysis. Since rowing is a sequential movement with a straightforward relationship between velocity and power, we opted for an LSTM network, as such networks have been proven to yield successful results for sequential motions like gait [36].

The LSTM network was able to reconstruct the forces and powers generated by the rowers with an overall mean absolute error smaller than 5% in ergometer and on-water rowing. Averaging over multiple cycles, justified by the repetitive and highly repeatable movements of rowing [47], brought the average error down to below 1%. Moreover, the individual rowers’ techniques, characterized by peak timing, mean to peak, and work ratio, were identified by the LSTM network with an error smaller than 4%. Finally, inter-individual differences in rowing technique were identified with an accuracy above 90%, except for the trunk power work ratio during ergometer rowing. The proposed method was thus able to quantify, with good accuracy, variables that are important for the evaluation of rowers’ performance [1,13] and techniques [9,12,17].

The estimation of the curve was accurate enough to identify fine details in the rowers’ techniques that could be addressed in training. For instance, in Figure 3A, the rowers on the ergometer show different strategies in terms of their arm power development, with subject 9 showing an early peak, followed by a session at negative power. This kind of curve is seen as a technical fault [1,48] since it means that some of the power generated by the rowers is absorbed by the arms instead of being transferred to the handle or oars. For this particular rower, the proposed method would facilitate the delivery of targeted feedback to correct the identified technical fault (i.e., a reduction in negative power, as highlighted on a monitor in front of the rower) while also providing objective metrics that the trainer can track over time (e.g., watts of negative power, ideally tending to zero). Real-time feedback has been proven to be helpful in boat and ergometer rowing [11,48,49], and the possibility of having targeted feedback with minimal equipment for both ergometers and boats would greatly benefit trainers and athletes.

The accuracy of body segment power estimation was lower than that of force estimation, particularly in the case of arm power, which was the segment with the lowest power production and presented the largest relative error. This result was expected since the forces are quantities that can be measured directly, while body segment power calculation is based on several assumptions combining multiple measurements. For instance, differences in sensor placement between subjects might lead to over- or underestimation of arm length and velocity, creating noise in power computation but not in the force measurements. We also identified additional sources of noise that limit the performance of our LSTM network. In the first place, the forces that we measured were unidirectional, but in reality, rowers can also exert forces in the vertical and lateral directions, which would affect the behavior of the boat and ergometer but not be captured by the sensors. Other sources of noise, while limiting the performance of the LSTM network on a cycle-by-cycle basis, could be mitigated when averaging the curves over multiple cycles. In particular, these sources include environmental interactions with water and wind, which might change within a single session [50], or oscillations of the ergometer along the rail. For instance, the average speed of the ergometer is obviously zero, but there might be cycle-by-cycle differences where the center of mass of the system (i.e., the rower plus the ergometer) moves a few centimeters backward or forward. These cycle-by-cycle variations make the model, in its current state, unsuitable for precisely identifying instantaneous changes in someone’s technique. However, given the repeatability of the rowing gesture [47], data averaged over a few cycles will provide important information regarding technique and performance progress over time. Some quantities could be more sensitive than others to these errors, such as the T2P of a curve with a large plateau, where small changes can have a larger effect on the peak position (e.g., for ergometer subject 3, leg power T2P exhibited less accuracy in discriminating between rowers). While the current method may not be accurate enough to discriminate between subjects with very similar techniques, the errors in identifying technical determinants were smaller than the variability observed among subjects employing different techniques. Indeed, the technical parameters used for describing rowers’ techniques showed good agreement between the measured and estimated sequences. When comparing the techniques between pairs of rowers, the determinants based on the estimated sequences had an accuracy of 93%, meaning legs–trunk–arms sequencing could be accurately estimated and used to inform crew selection based on the similarity of force and power profiles [16].

The accuracy of force and power estimation was much lower in the leave-one-out validation, with great variability between subjects. Considering the small number of subjects, this result was expected. Indeed, the rowers included in the study exhibited great variability in height, weight, and technique and might lie outside of the distribution of the small training set. In future research, more data should be collected to improve generalizability to larger populations of rowers [1]. In particular, the current sample was composed of only male rowers with similar ages and comparable skill levels and techniques [1]. Extending the analysis to women and younger or older rowers is a necessary step to include a variety of body types in the training set. Furthermore, the inclusion of data obtained from unskilled rowers (e.g., beginners or rowers on gym ergometers) could help the model learn specific mechanics behind the rowing movement that would be underrepresented in data collected from skilled rowers. The inclusion of these data might lead to the development of a computational tool capable of predicting the forces, powers, and technical profiles of any rower, whatever the rowing condition. Nonetheless, even in the current state, with a network trained on a small data sample corresponding to an average rowing club, this method shows promising results, allowing rowers’ techniques to be predicted in different settings, provided they had been previously evaluated once while using the complete biomechanical setup. Building on these promising results, future research should explore the feasibility of estimating force and power exclusively through video data. Such methods have already been successfully implemented for more complex movement types [25], and their application to rowing, particularly in the controlled environment of ergometer rowing, appears highly plausible.

Overall, the proposed method should significantly reduce the costs of performance analysis. Equipping an ergometer with only cable sensors could reduce costs by more than half, with video-based systems offering the potential for even greater savings. Similarly, while the cost of equipping a rowing boat currently exceeds EUR 2000, a set of IMUs and GPS devices can be acquired for less than half this cost, offering the advantage of being easily transferrable between rowers and boats.

To conclude, equipping rowing boats and ergometers with force and displacement sensors can be costly and time-consuming. The proposed method only requires the application of IMUs or cable sensors (ideally, for indoor rowing, these could also be replaced by video motion captures) and can quantify rowers’ performance and techniques with good accuracy.

## Figures and Tables

**Table 1:** List of features included in the LSTM network for boat and ergometer cases.

Input Features	Ergometer	Boat
Oar angular velocity	−	+
Boat horizontal velocity	−	+
Boat acceleration	−	+
IMU thoracic horizontal velocity WRT boat	−	+
IMU lumbar horizontal velocity WRT boat	−	+
IMU pelvis horizontal velocity WRT boat	−	+
IMU thoracic 1PCA ^1^ acceleration	−	+
IMU lumbar 1PCA ^1^ acceleration	−	+
IMU pelvis 1PCA ^1^ acceleration	−	+
IMU thoracic 1PCA ^1^ angular velocity	−	+
IMU lumbar 1PCA ^1^ angular velocity	−	+
IMU pelvis 1PCA ^1^ angular velocity	−	+
Average boat speed in the previous cycle	−	+
Max boat speed previous cycle	−	+
Height of rower	+	+
Weight of rower	+	+
Duration of the previous drive	+	+
Duration of current drive	+	+
Position of handle	+	−
Position of chest	+	−
Position of seat	+	−
Velocity of handle	+	−
Velocity of arms	+	−
Velocity of trunk	+	−
Velocity of seat	+	−
Acceleration of handle	+	−
Acceleration of trunk	+	−
Acceleration of legs	+	−
Maximum handle speed in the previous cycle	+	−
Time elapsed: maximum handle speed to beginning the current cycle	+	−

^1^ First principal component.

**Table 2:** LSTM performance. *MAE* and *cMAE*, absolute and normalized, are computed for the cycles in the validation set. Results reported for the leave-one-out validation (LOOV) consist of the mean errors over all subjects.

		*MAE*	*cMAE*	*MAE* Norm	*cMAE* Norm	LOOV *MAE*	LOOV *MAE* Norm	LOOV *cMAE*	LOOV *cMAE* Norm
Ergometer	$F^{f,x}$	19.15 N	2.67 N	3.06%	0.43%	66.20 N	10.40%	58.74 N	9.22%
	$F^{h,x}$	17.69 N	2.40 N	2.87%	0.39%	47.73 N	7.63%	39.53 N	6.32%
	$P^{h,x}$	37.04 W	5.44 W	2.96%	0.43%	95.81 W	7.69%	78.55 W	6.30%
	$P^{a}$	8.87 W	1.77 W	4.40%	0.88%	26.26 W	13.24%	22.37 W	11.23%
	$P^{t}$	14.50 W	1.68 W	2.88%	0.33%	35.64 W	7.05%	28.43 W	5.55%
	$P^{l}$	18.07 W	4.20 W	3.05%	0.71%	59.43 W	10.20%	57.79 W	9.07%
Boat	$F^{f,x}$	22.89 N	3.66 N	3.58%	0.57%	72.80 N	11.54%	65.94 N	10.49%
	$F^{h,x}$	23.15 N	4.13 N	3.33%	0.59%	61.40 N	8.91%	52.71 N	7.67%
	$P^{h,x}$	34.76 W	5.14 W	3.55%	0.52%	110.17 W	11.29%	97.48 W	10.03%
	$P^{a}$	20.52 W	3.13 W	8.25%	1.26%	60.89 W	25.15%	53.43 W	22.39%
	$P^{t}$	19.01 W	5.38 W	5.06%	1.43%	46.89 W	13.19%	39.48 W	11.25%
	$P^{l}$	27.26 W	4.91 W	5.64%	1.02%	67.81 W	14.05%	60.8 W	12.43%

**Table 3:** Mean ± standard deviation and mean absolute error (*MAE*) over all the cycles in the validation dataset for the technical determinants time to peak (T2P), mean to peak (M2P), and work ratio (WR).

		T2P (% Drive Phase)	T2P *MAE*	M2P (%)	M2P *MAE*	WR (%)	WR *MAE*
Ergometer	$F^{f,x}$	0.05 ± 1.59	0.97	−0.25 ± 1.09	0.86	0.11 ± 2.93	2.07
	$F^{h,x}$	0.02 ± 1.69	1.06	−0.16 ± 1.03	0.75	−0.08 ± 3.01	2.18
	$P^{h,x}$	0.00 ± 1.61	0.96	−0.13 ± 1.06	0.80	−0.15 ± 2.92	2.04
	$P^{a}$	−0.07 ± 1.07	0.48	0.04 ± 0.78	0.59	0.33 ± 3.55	2.25
	$P^{t}$	−0.02 ± 1.44	0.42	−0.05 ± 0.63	0.47	−0.12 ± 3.45	1.51
	$P^{l}$	0.23 ± 1.92	0.80	−0.12 ± 0.65	0.48	0.15 ± 4.30	2.25
Boat	$F^{f,x}$	0.31 ± 3.45	2.67	0.17 ± 1.39	1.15	0.59 ± 6.15	4.88
	$F^{h,x}$	0.20 ± 3.49	2.39	−0.16 ± 1.11	0.89	0.49 ± 5.99	4.13
	$P^{h,x}$	0.11 ± 1.72	1.22	0.25 ± 0.92	0.74	0.48 ± 3.12	2.35
	$P^{a}$	−0.34 ± 1.17	0.88	0.44 ± 1.64	1.26	−0.28 ± 4.17	3.15
	$P^{t}$	−0.06 ± 0.83	0.57	0.55 ± 0.92	0.80	0.29 ± 2.37	1.89
	$P^{l}$	0.26 ± 2.27	1.54	−0.19 ± 1.28	0.96	0.84 ± 4.92	3.55

## Data Availability

Data will be made available upon request. Code available at: https://github.com/loPitto/LSTM-scull-ergo (accessed on 4 January 2025).
